# Implementation Fidelity and Theory-Informed Dose Effects of a Teen Pregnancy Prevention Program for Native American Youth

**DOI:** 10.1007/s11121-023-01501-9

**Published:** 2023-05-16

**Authors:** Rachel A. Chambers, Christopher Kemp, Abagail Edwards, Summer Rosenstock, Angelita Lee, Laura Pinal, Etheline Cosen, Francene Larzelere, Lauren Tingey

**Affiliations:** 1 Johns Hopkins Center for American Indian Health, Johns Hopkins Bloomberg School of Public Health, 415 North Washington Street, Baltimore, MD 21231 USA; 2 Johns Hopkins Center for American Indian Health, Johns Hopkins Bloomberg School of Public Health, 102 General Crook Street, Fort Apache, AZ 85926 USA

**Keywords:** Process evaluation, Teen pregnancy prevention, Native American, Implementation, Sexual health

## Abstract

In 2019, Native youth had the highest rate of teen pregnancy of all racial/ethnic groups. “Respecting the Circle of Life” (RCL) is one of the first evidence-based teen pregnancy prevention programs for Native teens and there is interest in replicating the program across tribal communities. To inform replication, it is important to consider process data including quality, fidelity, and dosage as these may all moderate impact of the program. Participants were Native youth aged 11–19 and a trusted adult. This study includes participants randomized to the RCL program only (*N* = 266). Data sources include independent observations, facilitator self-assessments, attendance logs, and self-report assessments completed by enrolled youth at baseline and 3 months post assessment. Data was compiled and summed by cohort. Dosage was number of minutes participating in activities separated by theoretical constructs. Linear regression models were utilized to assess moderation of the effects of the intervention dosage on outcomes of interest. Eighteen facilitators delivered RCL. One hundred eighteen independent observations and 320 facilitator self-assessments were collected and entered. Findings indicate RCL was implemented with high fidelity and quality (4.40 to 4.82 out of a 5-point Likert scale; 96.6% of planned activities completed). Dosage was high with an average completion of 7 out of 9 lessons. There was no association between theoretical construct dosage and outcomes of interest. Overall, this study indicates RCL was delivered with high fidelity, quality, and dosage in this trial. This paper informs future replication of RCL and provides support for hiring paraprofessionals from the local community as facilitators, delivering the RCL to peer groups of the same age and sex, delivering the RCL with short duration and high frequency, and encouraging youth to attend all RCL lessons, but continue to serve youth who have missed one or more lessons.

## Introduction

The teen birth rate in the USA has declined 73% in the past three decades (Martin et al., 2019, 2021); however, at 16.7 births for every 1000 females aged 15–19, it is still higher than many other high-income countries including Canada and the UK (World Bank, n.d.). Further, racial/ethnic groups, including Native American (Native) youth, continue to be disproportionately impacted by teen pregnancy. In 2019, Native youth had the highest rate of teen pregnancy, a rate over twice that for non-Hispanic white youth (29.2 vs. 11.4), and were one of the only racial/ethnic groups who did not experience a decline in teen pregnancy rates between 2018 and 2019 (Martin et al., 2019, 2021). Despite these stark disparities, few teen pregnancy prevention programs that are contextually and culturally appropriate for Native youth have been evaluated, proven effective, and disseminated across Native communities.

To respond to the disparity in teen pregnancies and address the lack of Native-specific programming, the Johns Hopkins Center for American Indian health in partnership with a tribal community in the Southwestern United States developed the “Respecting the Circle of Life” (RCL) teen pregnancy prevention program (Chambers et al., 2016; Tingey et al., 2015). The RCL program was adapted from the evidence-based HIV risk reduction intervention, Focus on Youth (FOY) + ImPACT program (Stanton et al., 1996) rooted in the Protection Motivation Theory (PMT). The PMT posits that behavior is a product of balancing negative outcomes of a maladaptive behavior (e.g., unprotected sex) with the ability to complete a protective behavior (e.g., using a condom) (Rogers, 1983). During the adaptation of the program, cultural experts advised on inclusion of cultural content (which was limited but included discussion about leadership and goals) and adaptations to ensure the program was context specific (see Chambers et al., 2016), The RCL program was implemented and evaluated through a randomized controlled trial (RCT) funded by the Office of Population Affairs (Tingey et al., 2017) with Native youth aged 11–19. This grant was a part of the national, evidence-based teen pregnancy prevention grant program that funds diverse organizations to evaluate innovative and/or scale-proven programs to prevent teen pregnancy across the USA. Data from this trial provided evidence that the RCL program prevents risk factors across essential domains critical to the prevention of teen pregnancy among Native youth. Specifically after completing the RCL program, RCL participants were less likely to intend to have sex and more likely to intend to use a condom and reported higher condom use self-efficacy as well as contraceptive use self-efficacy compared to control participants (Tingey et al., 2021a). Further, participants in RCL had lower intention to use alcohol and drugs post intervention compared to control participants (Tingey et al., 2021b).

Given that RCL is one of the first programs proven efficacious at reducing risk for teen pregnancy among Native youth through a rigorous trial, efforts are now being made to disseminate the program to tribal communities around the USA. These efforts to disseminate the RCL program, in addition to the burgeoning field of implementation science, have brought to light a need to conduct a process evaluation of the RCL trial. Process evaluations generally explore the implementation, delivery, and setting of an intervention. These evaluations help researchers better understand the results of the outcomes of intervention trials and guide program implementers in replication efforts (Saunders et al., 2005). Further, a better understanding of the process of implementing interventions may highlight necessary refinements to intervention delivery mechanisms or content to improve reach or impact and they can be used during replication to refine intervention delivery (Linnan & Steckler, 2002).

It is important in a process evaluation to measure and ensure fidelity and quality of intervention delivery as this variable may moderate the relationship between an intervention and its outcomes (Carroll et al., 2007). In addition to fidelity and quality, impact of dose is among one of the most important aspects of implementation and is important to consider when designing, testing, and scaling behavioral interventions (Voils et al., 2012). Dose can be defined by frequency, amount, and duration of a program (Manojlovich & Sidani, 2008; Voils et al., 2012). Duration, which refers to the length of time in which a program is delivered as well as frequency, or how often contact is made over time, can impact the third aspect of dosage, amount, as the latter two components of dosage may influence participant burden, adherence, and ultimately the amount of intervention received by participants (Kardas et al., 2013; Voils et al., 2012). In this trial, RCL frequency was high and duration was short but varied across cohorts (Tingey et al., 2017). In other iterations of FOY, frequency has been lower and duration longer (Chen et al., 2010; Stanton et al., 1995; Stanton et al., 1996). Thus, in this paper, we aim to explore if higher frequency and lower duration significantly impacted adherence as well as amount of intervention received.

The amount of intervention received by participants may significantly impact outcomes, and thus, it is important before scaling interventions to understand the relationship between intervention amount and outcomes. Assessing amount of intervention received can be calculated in many ways including minutes of intervention, minutes of “core” components of an intervention, or minutes of theoretical content received. The latter is of specific interest for programs with a strong theoretical grounding such as RCL (Chambers et al., 2016, 2018; Stanton et al., 1995). RCL was developed through qualitative focus groups guided by the PMT. Further, a previous study found among Native youth, the five PMT constructs of extrinsic rewards, response efficacy, severity, vulnerability, and intrinsic rewards were all associated with condom use intention (a primary outcome of the RCL trial) (Chambers et al., 2018). Thus, one would hypothesize that full dosage (amount in min) of these five constructs may moderate impact of RCL on condom use intention and potentially other primary outcomes. A better understanding of how the dosage of each PMT construct impacts primary outcomes can help to refine RCL and inform program implementers as to how to implement RCL, specifically whether or not to put additional effort into ensuring youth receive all RCL content.

The goal of this paper is to first describe the implementation of the RCL program as it was delivered through the randomized controlled trial conducted between 2015 and 2020. Second, we aim to explore dosage and how the specific construct of dosage, amount of intervention received, is related to the trial’s primary outcomes. As interest in replicating RCL in other tribal communities increases, it is imperative to provide the results of this analysis to determine how communities can enhance RCL’s effectiveness, feasibility, and sustainability during replication efforts (Neta et al., 2015; Peters et al., 2014). Therefore, findings will directly inform future replication of the RCL program and potentially implementation of other teen pregnancy prevention programs in Native communities.

## Methods

### Study Design

This study was a randomized controlled trial. Participants were Native youth aged 11–19 and a parent or trusted adults (TAs). Dyads were randomized 1:1 (stratified by youth sex and age) to the intervention (RCL) or control condition immediately before completing the baseline assessment. All participants completed a baseline assessment, as well as a 3-month post assessment as a part of this evaluation. All participants were also enrolled in the process evaluation. This paper describes recruitment for the overarching trial in addition to implementation and outcome data specific to the intervention (RCL) group.

### Recruitment and Enrollment Procedures

Youth were recruited through multiple venues to enroll in the study. Data was not collected on how participants heard or were referred to the study, but primary recruitment efforts included the following: (a) posting flyers around the community (at the local grocery store, in schools, etc.), (b) presenting information and providing flyers at schools/parent-teacher conferences, (c) presenting information about the study at local clinics, (d) through social networks (many recruitment staff knew families with youth in the target age range and reached out to them), (e) through word of mouth (past participants would tell others about the study), and (f) announcements on the local radio station and print ads in the local paper. Potential participants or parents/guardians of potential participants who were interested in the study contacted the study office and spoke with a study staff member who completed an initial contact form. The study staff member then reviewed a recruitment form and, if eligible and interested, completed informed consent (if the youth was ≥ 18) or parental permission/assent (if youth was a minor < 18). After the youth was enrolled, they were asked to identify a TA to enroll with them. The TA could be enrolled anytime between the time of youth enrollment and the completion of the parent-youth lesson. This allows for flexibility and may aid in parent engagement.

### Description of the Respecting the Circle of Life Program Delivery

RCL is delivered through peer group lessons followed by a parent-youth lesson. The peer group component consisted of eight educational lessons, each lasting 90–120 min, delivered by two trained Native paraprofessionals to self-selected same-sex peer groups of 8–10 Native teens. The eight lessons were delivered once per day during an 8-day summer basketball camp. (Note while the camp primarily offered basketball as the non-study-related activity, other activities were offered to youth who did not enjoy basketball such as drawing, painting, arts and crafts, and photography.) The youth-parent component was one educational lesson lasting 90–120 min delivered by a trained Native paraprofessional who also taught the peer group lessons within 3 months after camp to the youth participant and enrolled TA together in their home. On the first day, youth were brought to the gym and asked to find a group of their friends/peers that were similar in age/grade and the same sex and go with that group to a number on the wall (the numbers were used to track group progress). Study staff then went to each group to ensure participants were of similar age (not more than 3 years between the oldest and youngest) in each group. Groups that were too large (> 12 youth) were separated. Groups were then assigned the morning or afternoon RCL lesson, the opposite of which they spent playing basketball or completing other activities as listed previously. The RCL study was conducted over three cohorts (cohort 1: July 2016, cohort 2: July 2017; cohort 3: June 2018). For cohort 1, RCL was delivered for 8 consecutive days (Friday–Friday). For cohorts 2 and 3, RCL was delivered for 8 consecutive weekdays with a break over the weekend (Weds–Friday and Monday–Friday).

The RCL program was delivered by two facilitators who were trained in the RCL program and PMT model. To identify facilitators, we advertised widely in the local community for applicants and met with current staff to discuss the opportunity. The local leadership team interviewed applicants/discussed the position with current staff and determined their fit for the position based on (1) cultural fit, (2) reliability, and (3) past teaching experience and/or ability to work with youth. All facilitators attended an initial week-long (40 h) RCL training. At the training, each facilitator was paired up with a co-facilitator who they would work with throughout the implementation of RCL. They then completed “co-facilitator worksheets” for each lesson indicating their role and their co-facilitator’s role on each activity within a lesson. They then attended an average of eleven, 1-h meetings with the trainer and their co-facilitator (these were conducted in person or via phone) where they roleplayed a lesson and received feedback. In total, facilitators completed on average 51 h of initial training. Finally, all facilitators passed a comprehensive exam before teaching the program. This exam focused on curriculum content (e.g., “What are three styles of communication discussed with the youth?”), curriculum concepts (e.g., “Why do the youth guess and discuss statistics related to youth sexual behaviors?”), reproductive and sexual health knowledge (e.g., “Please label the male reproductive system parts”), and program implementation (e.g., “Who should attend the parent/youth session?”). The entire 51-h training for facilitators along with completion of the exam was conducted prior to implementation of each cohort. Thus, facilitators may complete the training up to three times. Each facilitator pair worked with two groups (morning and afternoon). These groups and facilitators remained the same throughout the 8-day camp. Each morning, the Curriculum Director met with the facilitators for 30 min to prepare for the lesson. Additionally, all facilitators attended a daily 30-min debrief inclusive of a review of how the lessons went, discussion of questions, and an overview of the next day. At times, one facilitator would teach a lesson alone. While rare, this did occur during two of the cohorts. The majority of facilitators were female, from the local tribal community, and all had a high school diploma (see Table 1). Female facilitators’ age ranged from 23 to 50 years and men ranged from 23 to 38 years.

**Table 1:** Facilitator characteristics

Total number of facilitators	18
# cohorts taught by facilitator	
Taught only 1 cohort	8
Taught 2 cohorts	5
Taught all 3 cohorts	5
Facilitator age (mean, sd)	
Overall	30.7 (8.07)
Female	31.9 (8.62)
Male	29.3 (6.34)
Facilitator sex	
% Female	13 (72.2%)
Ethnicity	
Local community	16
Native American, not Apache	1
White	1
Facilitator education	
High school/GED	61.1%
Some college/college degree	38.9%
Average exam score, *m* (sd)	86.2% (11.4)

Within 3 months following the last peer group lesson, facilitators delivered the 9th, parent-youth lesson to the youth to the identified TA in the home or location of the youth/TA’s choosing. Although facilitators did all they could to ensure the parent-youth lesson included both the TA and the youth, if one was unavailable for a long period of time or refused to complete the lesson, the facilitator completed with just one member of the dyad.

#### Data Collection and Analysis

Data for the process evaluation was collected throughout implementation of RCL. Sources of data include the following: training tracking logs, participant tracking logs, independent observation forms, attendance records, and facilitator self-assessments. In order to conduct the implementation analysis, data was separated by cohort.

### Fidelity and Quality

Fidelity and quality were collected via two methods: (1) independent observations and (2) facilitator self-assessments. For peer group lessons, observations were conducted in person. Lessons were randomly selected to be observed with each classroom being observed at least once during the 8-lesson program. To observe lessons, a study staff member or student sat in on the class and, utilizing a written copy of the curriculum, completed a program observation form. For parent-youth lessons, lessons were randomly chosen to be audio recorded or directly observed. It was determined that in-person observations were sometimes inappropriate given the sensitive nature of the 9th lesson and the intimate environment in which it was delivered (participant’s home with youth and trusted adult); thus, the participant was given the option of in-person observation vs. audio. Audio recordings were uploaded to a secure webserver and listened to by an observer who completed an observation form. All observation forms were lesson specific and included information about the completion of each activity (was it completed and was it completed as intended or adapted) as well as the quality of program delivery. All observers completed a 2-h training in use of the observation form and the program manager reviewed all observation forms for completeness. Once reviewed, the data coordinator entered information from the observation forms into an Excel file. Data was aggregated and summarized to establish average fidelity and quality across all observations.

Facilitators completed a self-assessment immediately after completing each peer group lesson. To reduce staff burden, self-assessments were only completed for the first three parent-youth lessons completed. The facilitator self-assessment asked facilitators to provide information about quality and fidelity of the delivery of the program. All assessments were collected by the data coordinator who entered the information into an Excel file. Data was aggregated and summarized to establish average fidelity and quality across all observations.

### Dosage/Attendance

Attendance was collected via attendance sheets each day by study staff for the following: (1) attendance at camp, (2) attendance at basketball/gym/craft lesson, and (3) attendance at the RCL lesson. After each class, facilitators would provide attendance sheets to the manager who entered attendance in an Excel file. For the parent-youth lesson, facilitators recorded youth and TA attendance via lesson summary forms following a study visit. Once back at the office, study staff members entered attendance data for the parent-youth lesson into the Excel file. Dosage by lesson was compiled and summed to assess the percentage of youth attending each lesson as well as average number of peer group lessons (out of 8) attended by youth (see Table 2).

**Table 2:** Youth attendance by lesson and cohort

	**Overall**	**Cohort 1**	**Cohort 2**	**Cohort 3**	***p*-value**
Total Participants	266	80	115	71	
Lesson 1 peer group session, % Y (*n*)	94.36 (251)	93.75 (75)	96.52 (111)	91.55 (65)	0.346
Lesson 2 peer group session, % Y (*n*)	87.59	68.75 (55)	96.52 (111)	94.37 (67)	< .001
Lesson 3 peer group session, % Y (*n*)	78.20 (208)	62.50 (50)	90.43 (104)	76.06 (54)	< .001
Lesson 4 peer group session, % Y (*n*)	79.3 (211)	77.50 (62)	85.22 (98)	71.83 (51)	.081
Lesson 5 peer group session, % Y (*n*)	77.44 (206)	75 (60)	82.6 (95)	71.83 (51)	.191
Lesson 6 peer group session, % Y (*n*)	73.31 (195)	68.75 (55)	80.87 (93)	66.20 (47)	.049
Lesson 7 peer group session, % Y (*n*)	77.44 (206)	73.75 (59)	82.61 (95)	70.42 (50)	0.122
Lesson 8 peer group session, % Y (*n*)	75.94 (202)	76.25 (61)	79.13 (91)	70.42 (50)	.401
Lesson 9 parent/youth session, % Y (*n*)	82.3% (219)	96.3% (77)	74.8% (86)	78.9% (56)	.003
Average number of peer group lessons attended (out of 8), *m* (sd)	6.43 (2.09)	5.96 (2.29)	6.94 (1.62)	6.13 (2.37)	.002
Duration (# of weeks between lesson 1 to completion of lesson 9) *m*, ad	8.26 (5.81)	14.16 (4.02)	5.23 (4.28)	5.41 (2.61)	< .001

### Impact of Dosage on PMT Constructs and Intention Outcomes

#### Constuction of PMT Construct Dosage

To construct dosage data by PMT construct, prior to RCL implementation, each activity within a lesson was assigned a duration (e.g., 20 min) (see Table 3). Facilitators were asked to spend this amount of time on that activity. Each activity was then assigned one or more PMT construct(s) (e.g., activity 2, lesson 2 = vulnerability). These assignments were based on previous work conducted by the Focus on Youth + ImPACT program developer (Stanton et al., 1996). They were reviewed by the study leadership team to ensure agreement that the PMT construct was consistent with the activity. Total minutes across all 9 lessons for each PMT construct were added to establish a total dosage score for each construct (see Table 3). For each lesson attended by a youth (based on attendance data), the total minutes for each PMT construct for that lesson were included for that youth (e.g., if a youth attended lessons 1, 2, 3, and 6 only, their total dosage for PMT construct “severity” over the course of the program would be the sum of the “severity” construct for those lessons (25 + 40 + 15 + 30 or 110 min)).

**Table 3:** Lesson activities by PMT* constructs

	**Time (min)**	**PMT construct**							
		**None**	**Severity**	**Vulnerability**	**Internal rewards**	**External rewards**	**Self-efficacy**	**Response efficacy**	**Response costs**
**Lesson 1**									
2: RCL program overview	10	X							
3: Group cohesion (canyon/box/knot)	15	X							
4: Opening and closing rituals	15	X							
5: Group agreements	20	X							
6: Family tree	25		X	X	X	X			X
7: SPIRIT S + P	15	X							
* Dosage for PMT constructs in lesson 1*			25	25	25	25	0	0	25
**Lesson 2**									
2: Identifying the risk	20		X	X				X	
3: How risky is it?	20		X	X					
4: Am I invincible?	10			X					
5: What’s important to you?	10	X							
6: Ranking your values	15				X	X	X		X
7: To each their own: other’s values…	30				X	X	X		X
* Dosage for PMT constructs in lesson 2*			40	50	45	45	45	20	45
**Lesson 3**									
2: SPIRIT I	15						X	X	
3: Resources: how do I find out…	15		X	X			X	X	X
4: Pregnancy happens how?	75			X			X		
* Dosage for PMT constructs in lesson 3*			15	90	0	0	105	30	15
**Lesson 4**									
2: Communication with a trusted adult	20						X	X	X
3: Most teens are doing what?	15					X			
4: Condom demonstration	30						X	X	
5: Condom race	20						X		
6: SPIRIT R	25		X	X			X	X	X
* Dosage for PMT constructs in lesson 4*			25	25	0	15	95	75	45
**Lesson 5**									
2: SPIRIT IT	15						X	X	X
3: Communication games	20						X		X
4: Assert yourself	30						X	X	X
5: Sex: a decision for two	40		X	X	X	X	X	X	X
* Dosage for PMT constructs in lesson 5*			40	40	40	40	105	85	105
**Lesson 6**									
2: Showing you care	30		X		X	X	X	X	
3: STDs and unplanned pregnancy game	20			X					
4: Making the choice that’s right for me: contraception	55			X			X	X	X
* Dosage for PMT constructs in lesson 6*			30	75	30	30	85	85	55
**Lesson 7**									
2: STDS and unplanned pregnancy review	10		X	X				X	
3: Teen parent speaker	60								
4: Keeping my values	10		X	X					
5: Sticking to my decision roleplay	25						X	X	X
* Dosage for PMT constructs in lesson 7*			20	20	0	0	25	35	25
**Lesson 8**									
2: Making YOUR dreams come true	30		X	X	X		X	X	X
3: Obstacles to reaching goals	20		X	X	X		X	X	X
4: Identifying obstacles and concerns	10		X	X	X		X	X	X
5: Making a difference	10						X	X	
6: Buzz! Knowledge feud	25		X	X			X	X	
7: Pat on the back	15				X	X			
* Dosage for PMT constructs in lesson 8*			85	85	75	15	95	95	60
**Parent-youth lesson**									
Sexual Health 101	15		X	X					
Parent video	30	X							
Effective communication	20						X	X	X
Condom demo	10						X	X	
Talking with youth roleplays	20						X	X	X
Making dreams come true goal setting	15		X	X	X		X	X	X
* Dosage for PMT constructs in PY lesson*			30	30	15	0	65	65	55
***Total dosage by PMT construct***			**310**	**440**	**230**	**170**	**620**	**490**	**430**

^*^PMT protection motivation theory, the theoretical framework that underpins RCL

#### PMT Constructs and Intention Outcomes

Participants completed the Youth Health Risk Behavior Inventory (YHRBI), a self-report tool measuring sociodemographic variables, intentions, and seven PMT constructs at baseline and 3 months post intervention (Chambers et al., 2018). The YHRBI includes a 38-item questionnaire assessing the seven PMT constructs (self-efficacy, response efficacy, response cost, intrinsic reward, extrinsic reward, severity, and vulnerability) all scored on a 5-point Likert scale (see Table 1 in Chambers et al. (2018)). Additionally, the YHRBI included outcomes of interest as follows. Intention to have sex in the next year was measured by one question in which participants were asked if they intend to have vaginal sex in the next year. Response options included the following: Yes-definitely, Yes-probably, No-probably not, and No-definitely not. The scale was dichotomized so that “Yes-probably” and “Yes-definitely” were coded as 1 and other responses were coded as “0.” Condom use intention was measured by one question in which participants were asked if they would use a condom if they had sex in the next 6 months. Intention to use birth control and intention to get pregnant/get a girl pregnant in the next 6 months were also both asked with one question similar in structure to the condom use intention question. Response options for these questions included the following: yes, maybe, don’t know, probably not, and no. Again, these scales were dichotomized with “Yes” being coded a “1” and all other responses coded as “No” or “0.” Intention to wait to have sex until married was measured with the following question: “I want to wait until I’m married before I have sex.” Responses were a 5-point Likert scale from strongly agree to strongly disagree. Again, this was dichotomized with “strongly agree” and “agree” coded as “yes” or “1” and all other responses coded as “No” or “0.”

All youth participants completed the baseline assessment via ACASI on a laptop or tablet or via paper 0–3 days prior to randomization. Study staff supervised assessment completion and were available to answer questions as they arose. Three-month follow-up assessments were completed via ACASI or paper in the participants’ home or another place of their choosing approximately 3 months following completion of the parent-youth lesson (or the last camp lesson they attended if they did not complete the parent-youth lesson). All data were collected using a unique participant identification number with as few identifiers as possible.

### Statistical Analysis

We descriptively analyzed participant characteristics and PMT construct amount dosage over the course of their intervention participation, stratified by lesson completion (all vs. any missed). We then used multiple linear regression models with robust standard errors to assess for moderation of the effects of the intervention on PMT construct and intention outcomes in the treatment group, comparing participants with complete vs. incomplete PMT construct dosage. Beta coefficients were obtained for the continuous PMT construct outcomes and risk differences (RDs) (the absolute difference in the probability of the outcome between the full dosage vs. incomplete dosage participants) were obtained for the binary intention outcomes. PMT construct outcomes were standardized prior to analysis for ease of comparison. Covariates included PMT construct dosage (complete vs. incomplete) and adjustment for potential observed confounding by participant age (continuous, years) and sex (binary). The magnitude and statistical significance (*p* < 0.05) of the respective PMT construct dosage coefficients were assessed. Complete case analysis was used for all models. Analysis was performed in version 4.0.5 of R (1.13 Citing R | An Introduction to R, n.d.).

## Results

### Observations

Observations were conducted on 20% of peer group lessons (64 out of 240 lessons) and 25.2% of 9th parent-youth lessons (54 out of 210 lessons). Overall, reported fidelity and quality were high with ranges from 4.40 to 4.82 out of a 5-point Likert scale. Fidelity and quality did not vary significantly across cohorts (see Table 4).

**Table 4:** Fidelity and quality of RCL program implementation

	**Overall**	**Cohort 1**	**Cohort 2**	**Cohort 3**
**Observations**				
How well were activities explained^a^	4.82 (0.48)	4.91 (0.35)	4.80 (0.55)	4.7 (0.54)
Facilitator kept track of time^a^	4.78 (0.49)	4.91 (0.35)	4.72 (0.45)	4.62 (0.70)
Participants understand content^a^	4.56 (0.69)	4.77 (0.47)	4.34 (0.83)	4.52 (0.64)
Participants actively participated^b^	4.40 (0.81)	4.54 (0.69)	4.39 (0.86)	4.19 (0.74)
Facilitator was knowledgeable about the topic^b^	4.62 (0.68)	4.72 (0.63)	4.52 (0.76)	4.63 (0.63)
Facilitator was enthusiastic^b^	4.63 (0.65)	4.76 (0.48)	4.5 (0.82)	4.63 (0.56)
Facilitator had good rapport with participants^b^	4.75 (0.51)	4.82 (0.49)	4.70 (0.56)	4.77 (0.43)
**Fidelity facilitator self-assessments**				
% Activities completed	96.6% (1927)	97.9% (444)	95.8% (745)	97.3% (738)
% Activities adapted	7.0% (157)	11.2% (58)	4.8% (39)	6.4% (60)

^a^Scale: 1 = not well to 5 = very well

^b^Scale: 1 = not much to 5 = very much

### Facilitator Self-assessments

A total of 320 facilitator self-assessments (72 in cohort 1121 in cohort 2 and 127 in cohort 3) were completed and entered into the Excel data base. Across all self-assessments, facilitators were asked if they believed the youth were “bored” during the lesson; facilitators strongly agreed or agreed with this statement for 7.8% of the lessons. In total, the 320 facilitator self-assessment forms asked about completion as intended for 1995 intervention activities (457 in cohort 1, 779 in cohort 2, and 759 in cohort 3). Fidelity to these activities was high with 96.6% of activities completed and only 7.0% adapted. Two primary reasons were given for not completing the activity: “ran out of time” and “didn’t have the supplies.” Adaptations to the activities included adding additional examples, leaving out a portion of an activity (e.g., not using a visual), or reducing the length of an activity. For the parent-youth lesson, key adaptations included not conducting the condom demonstration with the parent but instead walking through the steps (see Table 4).

### Dosage/Attendance

Participants attended on average 6.43 or 80.4% of peer group lessons and 82.9% completed the 9th parent-youth lesson. Dosage varied across cohorts with cohort 1 having the lowest peer group attendance (5.96 in cohort 1 vs. 6.94 in cohort 2 and 6.13 in cohort 3) and the highest percentage of youth completing the parent-youth lesson (96.3% in cohort 1 vs. 74.8% in cohort 2 and 78.9% in cohort 3). There were also significant differences in time between the last peer group lesson and the 9th parent-youth lesson with cohort 1 having an average time between these lessons almost 3 times that of cohorts 2 and 3 (see Table 2).

### Dosage Amount and Outcomes

A total of 266 youth were included in the RCL program with 39.8% (*n* = 106) attending all lessons and therefore receiving full-minute dosage of all PMT constructs. The average age of youth was 13.26 (standard deviation (SD): 1.8), 52.3% were female, and 33.5% spoke a Native language. There were no significant differences in age, sex, or language across lesson completion strata. The average number of minutes received by youth of each PMT construct overall and by those who had complete vs. incomplete attendance can be found in Table 5.

**Table 5:** Baseline demographics and PMT* mean dosage by lesson completion

	**Overall**	**Incomplete PMT construct dosage**	**All PMT construct dosage**	***p*-value**
*N*	266	160	106	
***Sociodemographic characteristics***				
Age, years, mean (SD)	13.26 (1.80)	13.33 (1.77)	13.16 (1.85)	0.45
Female, *N* (%)	139 (52.3)	76 (47.5)	63 (59.4)	0.075
Speaks a Native language, *N* (%)	89 (33.5)	52 (32.5)	37 (34.9)	0.784
***PMT construct dosage***				
Severity, minutes, mean (SD)	247.97 (79.71)	206.88 (79.53)	310.00 (0.00)	< 0.001
Vulnerability, minutes, mean (SD)	357.12 (115.79)	295.59 (113.07)	450.00 (0.00)	< 0.001
Internal rewards, minutes, mean (SD)	185.39 (60.36)	155.84 (62.19)	230.00 (0.00)	< 0.001
External rewards, minutes mean (SD)	139.27 (43.03)	118.91 (45.16)	170.00 (0.00)	< 0.001
Self-efficacy, minutes mean (SD)	485.83 (166.53)	396.94 (162.08)	620.00 (0.00)	< 0.001
Response efficacy, minutes, mean (SD)	381.60 (138.32)	309.78 (137.34)	490.00 (0.00)	< 0.001
Response cost, minutes, mean (SD)	342.50 (111.19)	284.53 (110.09)	430.00 (0.00)	< 0.001

^*^PMT protection motivation theory, the theoretical framework that underpins RCL

Table 6 presents the relationship between each PMT construct dosage and PMT construct and intention outcomes at 3 months. No significant differences in PMT construct and intention outcomes were identified comparing participants with complete vs. incomplete PMT construct dosage.

**Table 6:** Relationship between complete dosage status and PMT* and intention outcomes (*n* = 266)

**PMT dosage completion**	**PMT outcomes**							**Intent outcomes**					
	**Severity**	**Vulnerability**	**Internal rewards**	**External rewards**	**Self-efficacy**	**Response efficacy**	**Response cost**	**Have SEX (in next year)**	**Have sex (in next 6 mo.)**	**Use condoms (in next 6 mo.)**	**Use contraception (in next 6 mo.)**	**Become pregnant (in next 6 mo.)**	**Abstain from sex until marriage**
	***β (95% CI)***	***β (95% CI)***	***β (95% CI)***	***β (95% CI)***	***β (95% CI)***	***β (95% CI)***	***β (95% CI)***	***RD (95% CI)***	***RD (95% CI)***	***RD (95% CI)***	***RD (95% CI)***	***RD (95% CI)***	***RD (95% CI)***
**Severity**	− 0.18 (− 0.45, 0.08)	− 0.01 (− 0.24, 0.22)	0.1 (− 0.13, 0.33)	− 0.04 (− 0.25, 0.17)	0.01 (− 0.22, 0.25)	0.02 (− 0.24, 0.28)	− 0.02 (− 0.29, 0.25)	− 0.05 (− 0.18, 0.08)	0.02 (− 0.05, 0.08)	0.03 (− 0.11, 0.17)	0.05 (− 0.77, 0.87)	0.01 (− 0.01, 0.04)	− 0.03 (− 0.16, 0.1)
**Vulnerability**	− 0.18 (− 0.45, 0.08)	− 0.01 (− 0.24, 0.22)	0.1 (− 0.13, 0.33)	− 0.04 (− 0.25, 0.17)	0.01 (− 0.22, 0.25)	0.02 (− 0.24, 0.28)	− 0.02 (− 0.29, 0.25)	− 0.05 (− 0.18, 0.08)	0.02 (− 0.05, 0.08)	0.03 (− 0.11, 0.17)	0.05 (− 0.77, 0.87)	0.01 (− 0.01, 0.04)	− 0.03 (− 0.16, 0.1)
**Internal rewards**	− 0.17 (− 0.43, 0.09)	− 0.16 (− 0.38, 0.07)	− 0.04 (− 0.26, 0.18)	− 0.06 (− 0.28, 0.15)	0.09 (− 0.14, 0.33)	0.15 (− 0.1, 0.41)	− 0.04 (− 0.31, 0.23)	− 0.05 (− 0.18, 0.08)	0.04 (− 0.02, 0.1)	0.03 (− 0.1, 0.17)	0.1 (− 0.75, 0.96)	0.01 (− 0.01, 0.03)	0.03 (− 0.1, 0.16)
**External rewards**	− 0.24 (− 0.5, 0.01)	− 0.07 (− 0.3, 0.15)	0.06 (− 0.16, 0.28)	− 0.13 (− 0.35, 0.09)	0.02 (− 0.22, 0.25)	− 0.01 (− 0.27, 0.25)	0.11 (− 0.16, 0.39)	0 (− 0.13, 0.14)	0.03 (− 0.03, 0.09)	0.04 (− 0.1, 0.18)	0.13 (− 0.74, 1)	0.01 (− 0.01, 0.03)	− 0.07 (− 0.2, 0.06)
**Self-efficacy**	− 0.18 (− 0.45, 0.08)	0.02 (− 0.21, 0.24)	0.09 (− 0.14, 0.31)	− 0.04 (− 0.25, 0.17)	0 (− 0.23, 0.23)	0 (− 0.26, 0.26)	− 0.03 (− 0.3, 0.24)	− 0.05 (− 0.19, 0.08)	0.01 (− 0.05, 0.08)	0.02 (− 0.12, 0.16)	0 (− 0.82, 0.82)	0.01 (− 0.01, 0.04)	− 0.05 (− 0.17, 0.08)
**Response efficacy**	− 0.18 (− 0.45, 0.08)	0.02 (− 0.21, 0.24)	0.09 (− 0.14, 0.31)	− 0.04 (− 0.25, 0.17)	0 (− 0.23, 0.23)	0 (− 0.26, 0.26)	− 0.03 (− 0.3, 0.24)	− 0.05 (− 0.19, 0.08)	0.01 (− 0.05, 0.08)	0.02 (− 0.12, 0.16)	0 (− 0.82, 0.82)	0.01 (− 0.01, 0.04)	− 0.05 (− 0.17, 0.08)
**Response cost**	− 0.18 (− 0.45, 0.08)	− 0.01 (− 0.24, 0.22)	0.1 (− 0.13, 0.33)	− 0.04 (− 0.25, 0.17)	0.01 (− 0.22, 0.25)	0.02 (− 0.24, 0.28)	− 0.02 (− 0.29, 0.25)	− 0.05 (− 0.18, 0.08)	0.02 (− 0.05, 0.08)	0.03 (− 0.11, 0.17)	0.05 (− 0.77, 0.87)	0.01 (− 0.01, 0.04)	− 0.03 (− 0.16, 0.1)

all *p*-values > 0.05

*RD* risk difference, *CI* confidence interval

^*^PMT protection motivation theory, the theoretical framework that underpins RCL

## Discussion

This study assessed implementation of the RCL program as part of a randomized controlled trial and sought to assess the three components of dosage: duration and amount as well as fidelity and quality. Results suggest the program was implemented with high quality and fidelity and provides guidance for other tribal communities planning to implement RCL. Additionally, we found no relationship between PMT dosage amount and intention outcomes, suggesting that perfect attendance was not imperative for the intervention to have positive effects.

The high fidelity and quality of the intervention as it was delivered, namely the delivery by local paraprofessionals from the community, are promising for replication and sustainability in Native communities. These results indicate high fidelity to the intervention does not require facilitators to have an advanced degree or formal teaching experience outside of training in the RCL curriculum. While likely no formal education is required to facilitate RCL, sufficient training including at the minimum a week-long training in addition to extensive practice through roleplays is necessary. Replication efforts for RCL should include ample budget and time to provide this training and time to practice.

It is encouraging to observe the high rates of fidelity and quality reported by facilitators through the facilitator self-assessments were mirrored in the observation forms, which were completed by independent observers. Often, there are concerns about self-reported fidelity because facilitators may be influenced by social desirability and, thus, may provide inflated reports of fidelity (Gresham et al., 2000). We did not see this in our data. Future implementation of RCL should continue to include fidelity monitoring, and our results indicate it may be sufficient to collect this data via self-report by facilitators. A limitation to this analysis was that we were unable to assess how characteristics of the facilitator impacted outcomes. We would hypothesize based on previous research that youth whose facilitators were culturally matched (Native) and who had a higher education and/or experience teaching would report greater positive impact of the RCL program. Future studies should explore facilitator characteristics including cultural match, education, gender, and age on outcomes.

With an average completion of 80% of lessons, findings also suggest delivering RCL with increased frequency but reduced duration, specifically, through an 8-day basketball camp followed by a home-based parent-youth lesson, results in high levels of intervention received (amount). While we did not collect data on the impact of the retention efforts that we made (see Tingey et al. (2017) for more details on these retention efforts) including daily phone calls, transportation services to camp, and monetary and non-monetary incentives, these were assumed, in addition to the delivery design contributed to the high level of dosage observed. If camps are not available, it may be possible to integrate the RCL program through after-school programs, in schools, and/or in partnership with community-based groups during school breaks.

Given the difference in attendance (and overall amount of intervention received) between the cohorts, specifically that in cohort 1, the two lessons taught on the weekend (lessons 2 and 3) had substantially lower rates of attendance compared to other cohorts; findings indicate future replication of RCL should avoid delivering lessons on the weekend and thus extend the 8 peer-lesson duration from 8 consecutive days to 10 days with a break on the weekend. Additionally, the significantly higher rate of parent-youth lesson completion in cohort 1 coupled with the significantly longer duration between the last peer group lesson and the parent-youth lesson indicates that spreading the parent-youth lesson out from the last peer group lesson may be advantageous as far as increasing level of completion of the parent-youth lesson. Due to the small sample size across cohorts and the lack of randomization for the timing of the intervention or cohort, we are unable to ascertain if the increase in time between the peer group lesson and the parent-youth lesson impacts outcomes in a positive or negative way. The impact of duration on outcomes should be assessed in future trials of RCL.

It is promising that we did not see significant variations in amount of intervention (specifically PMT construct amount in min) received by age or sex—suggesting the RCL program when delivered to youth of the same sex and age group is appealing and engaging for all youth aged 11–19. There was some hesitancy by facilitators to engage the younger youth in this program, however, based on the lack of differences in dosage across age combined with high fidelity across lessons indicates RCL is appropriate for this age group. This may be due to the fact that RCL provides opportunities for the facilitators to tailor the curriculum by sex and age—a key concept that may have contributed to the lack of differences by these characteristics. Many lesson activities that include examples of situations youth may find themselves in are not “one-size fits all.” There are multiple examples, and thus, the facilitator can choose one that seems to best reflect the sex and experiences (or age) of the group members. Regardless of why, that the RCL program seems to appeal to youth of all ages and sexes is promising as boys, particularly older boys, are often harder to engage and retain in programming (Walker et al., 2016). Further, as illustrated in Table 1, the majority of facilitators were female, and thus, most boy-specific groups were talked by mixed-sex facilitators (male + female). That there were no differences between boys and girls in dosage, despite being taught by females, is promising. We conclude that the delivery structure (small peer groups of same sex and age) for all teen pregnancy prevention programming in Native communities should be considered to maximize adherence/dosage amount, especially among this hard-to-reach subset of youth. Based on these results, it is suggested that RCL continue to be delivered in small peer groups of the same age and sex for maximum adherence. Additional studies are needed to better understand adherence with mixed-sex and/or mixed-age groups.

The lack of significance seen between primary outcomes and PMT dosage may be somewhat impacted by the large proportion of youth who received 100% of all PMT construct dosage amount or the small sample size. It most likely means that complete dosage of the program is not necessary to see positive impact on primary outcomes and is a strong contribution to the literature specific to teen pregnancy prevention in Native communities. This result is also promising for replication of the program in real-world settings as it may be assumed that unlike this research trial in which resources were not significantly constrained, in resource-constrained communities, it may be harder to attain the high level of attendance/dosage amount as seen in this trial. This analysis was limited in that we cannot ascertain what level of PMT construct dosage is optimal for the best outcomes, and future studies of RCL should aim to include a large enough sample size to conduct this analysis and/or randomly assign youth to certain amounts of the intervention.

## Conclusion

Overall, this study indicates the RCL program was delivered with high fidelity, quality, and dosage in this trial. This paper informs organizations that choose to replicate RCL should (1) employ paraprofessionals from the local community as RCL facilitators; (2) provide substantial training as well as technical assistance and tools to RCL facilitators; (3) ask facilitators to document quality and fidelity of their delivery; (4) deliver RCL to peer groups of the same age and sex; (5) deliver the RCL with short duration and high frequency, but avoid weekend program delivery (e.g., 2-week sports camp with parent-youth lesson delivered 1–5 months after); and (6) encourage youth to attend all RCL lessons, but continue to serve youth who have missed one or more lessons.

## Data Availability

The data utilized in this manuscript is owned by the tribal community with which this research was conducted. Thus, the data is not publicly available and any access to the data is at discretion of tribal authorities.
