# Correction: Implementation Fidelity and Theory-Informed Dose Effects of a Teen Pregnancy Prevention Program for Native American Youth

**DOI:** 10.1007/s11121-023-01623-0

**Published:** 2023-12-08

**Authors:** Rachel A. Chambers, Christopher Kemp, Abagail Edwards, Summer Rosenstock, Angelita Lee, Laura Pinal, Etheline Cosen, Francene Larzelere, Lauren Tingey

**Affiliations:** 1 Johns Hopkins Center for American Indian Health, Johns Hopkins Bloomberg School of Public Health, 415 North Washington Street, Baltimore, MD 21231 USA; 2 Johns Hopkins Center for American Indian Health, Johns Hopkins Bloomberg School of Public Health, 102 General Crook Street, Fort Apache, AZ 85926 USA

**Correction to: Prevention Science** **https://doi.org/10.1007/s11121-023-01501-9**

The article “Implementation Fidelity and Theory‑Informed Dose Effects of a Teen Pregnancy Prevention Program for Native American Youth”, written by Chambers, R.A., Kemp, C., Edwards, A., Rosenstock, S., Lee, A., Pinal, L., Cosen, E., Larzelere, F., and Tingey, L., was originally published electronically on the publisher’s internet portal on 16 May 2023 without open access. With the author(s)’ decision to opt for Open Choice the copyright of the article changed on 17 November 2023 to © The Author(s) 2023 and the article is forthwith distributed under a Creative Commons Attribution 4.0 International License, which permits use, sharing, adaptation, distribution and reproduction in any medium or format, as long as you give appropriate credit to the original author(s) and the source, provide a link to the Creative Commons licence, and indicate if changes were made. The images or other third party material in this article are included in the article’s Creative Commons licence, unless indicated otherwise in a credit line to the material. If material is not included in the article’s Creative Commons licence and your intended use is not permitted by statutory regulation or exceeds the permitted use, you will need to obtain permission directly from the copyright holder. To view a copy of this licence, visit http://creativecommons.org/licenses/by/4.0/.

The original article has been corrected.

